# The distribution and population structure of two macaque species in Xizang, China 

**DOI:** 10.3897/BDJ.14.e199726

**Published:** 2026-10-02

**Authors:** Lin Li, Hongbo Li, Chang Luo, Fanmiao Zhou, Zhonglian Wang, Qiuxia Wang, Zifa Zhao, Jiang Zhou

**Affiliations:** 1 School of Karst Sciences, Guizhou Normal University, Guiyang, China School of Karst Sciences, Guizhou Normal University Guiyang China; 2 Guizhou Forestry Foreign Cooperation and Industry Development Center, Guiyang, China Guizhou Forestry Foreign Cooperation and Industry Development Center Guiyang China; 3 Faculty of Science, Eberhard Karls Universität Tübingen, Tübingen, Germany Faculty of Science, Eberhard Karls Universität Tübingen Tübingen Germany; 4 School of Life Sciences, Guizhou Normal University, Guiyang, China School of Life Sciences, Guizhou Normal University Guiyang China

**Keywords:** new distribution records, primates, Arunachal macaque, white-cheeked macaque, rare and endangered species

## Abstract

Xizang, China, has complex alpine ecosystems and supports a diversity of primates. However, systematic survey data on the current distribution and population characteristics of the white-cheeked macaque (*Macaca leucogenys*) and the Arunachal macaque (*Macaca munzala*) in Xizang remain limited. To determine the spatial distributions and troop sizes of the two macaque species, line-transect surveys and camera-trap monitoring were conducted in Shannan City and Nyingchi City, Xizang, in 2025. The surveys recorded 11 troops of Arunachal macaques in Himalayan fir forests and forest-edge habitats at elevations of 2,000–3,000 m in Cona City, Shannan City and 10 troops of white-cheeked macaques in habitats, ranging from evergreen broad-leaved forest to mixed coniferous–broad-leaved forest at elevations of 1,395–2,700 m in Gedang Township and Damu Township, Medog County, Nyingchi City. Average troop size was approximately 14 individuals for the Arunachal macaque and 17 individuals for the white-cheeked macaque. Troops of both species included adults, subadults and infants and the presence of infants indicated normal reproductive activity. This study provides the first systematic documentation of the current distribution ranges, habitat preferences and social structure of these two macaque species in Xizang, thereby providing baseline data for subsequent conservation assessments and management decisions.

## Introduction

More than 60% of primates worldwide are threatened by habitat loss, climate change and human activities and accurate assessments of their populations and distributions are a prerequisite for developing conservation strategies (Estrada et al. 2017; Songtao et al. 2026). The Xizang Autonomous Region occupies the main part of the Qinghai–Xizang Plateau. Its enormous elevational range has produced a complete vertical vegetation zonation from subtropical montane forest to alpine meadow, providing diverse habitats for multiple primate species (Jiang and Bleisch 1996; Yu et al. 2016; Zhu et al. 2026). Rare species recorded in this region include the Arunachal macaque (*Macaca munzala*, hereafter SXM), white-cheeked macaque (*M. leucogenys*, hereafter WCM), black-and-white snub-nosed monkey (*Rhinopithecus bieti*), Tibetan macaque (*M. thibetana*), Xizang rhesus macaque (*M. mulatta vestita*), Himalayan grey langur (*Semnopithecus schistaceus*), Assamese macaque (*M. assamensis*) and Shortridge's langur (*Trachypithecus shortridgei*), which was first recorded there in 2025. These species differ markedly in ecological requirements and distribution patterns and their conservation status varies (Wei et al. 2025; Ren et al. 2026). However, whether the two recently discovered Macaca species in the region — the Arunachal macaque and the white-cheeked macaque — have stable distributions within Xizang has long been disputed, severely constraining population assessments and the development of conservation strategies.

The Arunachal macaque (*Macaca munzala*) and white-cheeked macaque (*M. leucogenys*) are two new macaque species described successively from the eastern Himalayas in the 21^st^ century. The Arunachal macaque was named in 2005 and is characterised by a pale yellow crown patch, centrally whorled black hair and dark stripes above the eyes (Sinha et al. 2005); subsequent surveys confirmed that its distribution extends into Bhutan (Sarania et al. 2017; Tobgay et al. 2019). The white-cheeked macaque was formally described and published as a new species by Li et al. in 2015, based on photographs obtained through direct observations and camera traps in Medog, Xizang and morphological comparisons with potentially sympatric macaques (*M. mulatta*, *M. thibetana*, *M. assamensis* and *M. munzala*). It is characterised by greyish-white to white pelage on the cheeks and chin (Li et al. 2015). The original paper suggested that the species might also occur in Pomi, Zayu and other areas, but this had not been verified in the field. The type localities of both macaques lie in the eastern Himalayas within China — the former in southern Xizang and the latter in Medog County, south-eastern Xizang. Nevertheless, the suggestions in the original literature that stable, self-sustaining populations of the two species occur in Xizang have long lacked conclusive field evidence (Sinha et al. 2005;Li et al. 2015). Here, “stable distribution” is defined as continuous or regular records of a species within a specific area, together with the presence of populations capable of carrying out essential life activities, such as reproduction and foraging. This long-standing distributional uncertainty has not only left IUCN Red List assessments without a reliable basis, but has also deprived habitat conservation planning, transboundary population management and community-based conservation measures of a scientific spatial foundation. Confirming the current distributions of these two macaque species in Xizang is, therefore, an urgent prerequisite for assessing their degree of threat and developing effective conservation strategies.

In view of these critical information gaps, systematic surveys of the Arunachal macaque and white-cheeked macaque were conducted in south-eastern Xizang from April to September 2025. This period coincides with the post-parturition lactation and infant-following stages of Macaca species, when infants are readily identifiable and reproductive parameters can be recorded more easily (DP et al. 2026). At the same time, rising temperatures, abundant food, frequent macaque activity and good transect accessibility in Xizang help improve survey efficiency and data accuracy. By integrating automated camera-trap monitoring with walking transect surveys, this study aimed to determine whether stable populations of the two macaque species occur in Xizang and to obtain precise occurrence localities. It also sought to obtain preliminary baseline ecological information on their social structure, habitat types and habitat-use characteristics. The results provide essential baseline data for clarifying the geographic distribution boundaries of the two macaque species, assessing their current population status and developing science-based transboundary conservation strategies.

## Study Area and Methods

### Study Area 

The study area is located in south-eastern Xizang Autonomous Region, at the tectonic bend near the eastern end of the Himalayas, where deep incision by the Yarlung Zangbo River has formed a massive canyon. The terrain is high in the northwest and low in the southeast, with extreme vertical relief and a complete vertical zonation from alpine snow and ice to tropical monsoon forest at lower elevations. The study area encompasses Nyingchi City (Bayyi District and selected townships in Medog and Zayu counties) and the southern valley zone of Cona County, Shannan City (Fig. A in Fig. 1) extending from 91°57'–98°47' E and 26°52'–30°40' N. The region is a humid montane canyon forest zone on the south-eastern slope of the Himalayas and is influenced by the southwest monsoon from the Indian Ocean. Mean annual precipitation is 800–1,400 mm and is concentrated from May to September. The mean annual temperature is 15.8°C in the valleys (1,500 m), with a January mean of 7.2°C and 6.3°C on the ridges (3,800 m), with a January mean of −2.1°C. Sampling sites were restricted to elevations of 1,500–4,000 m. The lower and middle montane belt at 1,500–2,500 m supports monsoon evergreen broad-leaved forest, dominated by *Castanopsis hystrix*, *Machilus nanmu* and *Altingia chinensis*. The middle montane belt at 2,500–3,800 m supports mixed coniferous–broad-leaved forest dominated by *Tsuga chinensis*, *Picea likiangensis* and *Cyclobalanopsis glauca*, with dense understorey thickets of *Fargesia spp. and Rhododendron spp*.; canopy closure in primary forest exceeds 0.85. One fixed monitoring transect was established at each 500-m interval along the elevational gradient and sampling points were georeferenced using a hand-held GPS unit (accuracy ≤ 5 m). Sites were selected on the basis of three criteria: (1) coverage of the valley zones with the highest frequency of historical records of the target species; (2) high forest-landscape connectivity (patch fragmentation index < 0.12), a straight-line distance of > 10 km from the nearest rural settlement and very low human disturbance; and (3) the presence of potential dispersal corridors formed by the lower Yarlung Zangbo River valley and southern low-mountain passages, facilitating detection of population distribution boundaries.

### Camera-Trap Deployment and Field Surveys 

Camera-trap monitoring and transect surveys were conducted concurrently. A total of 120 functional camera-trap stations were established using the “Yianweishi L810G Full-Network” infrared-triggered monitoring unit manufactured by Qingdao Yianweishi Technology Co., Ltd. (IP68 protection; equipped with infrared/LED illuminators and a passive infrared sensor; automatically triggers photographs or video when infrared signals are detected within the effective sensing range; suitable for scientific field surveys and long-term biodiversity monitoring). Camera stations were preferentially established near water sources, on animal trails and in accessible areas with clear signs of animal activity (tracks, feeding signs or faeces). Stratified sampling was used to cover major habitat types, including forest and shrubland, while a 1 km x 1 km grid constrained the overall distribution of stations to reduce bias (infrared cameras and transects are shown in the B section of Fig. 1). Each camera was secured to a tree trunk at a height of 1.5-2.0 m using a wear-resistant strap. This height reduced false triggers caused by low shrubs, splashed mud or water and small animals. The detection zone extended from the ground in front of the camera to approximately 3 m up the tree trunk, thereby covering some activity in the lower arboreal layer; missed detections of activity in the higher canopy were supplemented by concurrent transect surveys (Jiang and Bleisch 1996; Karanth and Nichols 1998; Luskin et al. 2023; Jiao 2024). The lens was kept horizontal or angled slightly downwards and obstructions in front of the camera were removed to reduce false triggers. The operating mode was set to “three consecutive photographs + a 10 s video”, with a trigger interval of 1 s and medium sensitivity. A straight-line distance of ≥ 500 m between adjacent stations ensured independence, consistent with Chinese specifications for camera-trap monitoring of wildlife (Swann et al. 2004; S et al. 2014; Wearn and Glover-Kapfer 2017; Tanwar et al. 2021). A single camera operating continuously for 24 h was defined as one effective camera-day and the effective detection radius at each station was approximately 3 m. From April to September 2025, a period of 183 days, the 120 stations operated effectively for a mean of 150 days after accounting for malfunctions and battery- or memory-card problems, yielding a cumulative sampling effort of 18,000 effective camera-days. For the same species photographed at the same station, two records separated by ≥ 30min were treated as two independent valid records, whereas consecutive detections separated by < 30min were combined as one record. This 30 min threshold follows the camera-trap data standard of O'Brien et al. (2003) and is also widely used in camera-trap monitoring of biodiversity in China.

Based on the independent records, two observers independently classified the species, sex and age class of animals in the photographs and videos. Species were identified from external morphology, pelage colour, facial features and body size; sex was determined from external genitalia, nipples, body size and facial sexual characteristics; and age was classified as adult, subadult or infant. Agreement between the two observers was evaluated using Cohen's κ (Cohen 1960): κ ≥ 0.75 was considered high confidence; values of 0.40-0.75 were considered moderate confidence and required review by a third observer; and records with κ < 0.40 were excluded or classified only as "undetermined." Individuals that could be reliably distinguished from natural markings — including facial contours, pelage patches, tail length, body size, conspicuous scars, a shortened tail, external genitalia or nipples—were identified individually (JM and DJ 2011; Li et al. 2015; Chang et al. 2018; Shen et al. 2025) and used to calculate the minimum number of individuals observed. Records for which individuals could not be identified reliably were used only in species-distribution and troop-level analyses.

To supplement the limited ability of camera traps to detect highly mobile individuals and macaque troops in blind spots and to cross-validate data reliability, 22 transects were established concurrently. Each transect was 5 km long and 30 m wide, extending 15 m on each side of the center line. Stratified random sampling covered habitat types, including forest, shrubland and exposed rock. A minimum distance of 500 m between transects prevented spatial overlap and transect routes avoided animal trails and human paths whenever possible to reduce detection bias. From April to September 2025, each transect was surveyed once per month, for a total of 132 surveys and an effective cumulative transect distance of 660 km (detailed survey effort by region and species is provided in Supplementary Table S1). Surveys were conducted 2-4 h after sunrise and 2-4 h before sunset to cover peak periods of primate activity (Swann et al. 2004; Li et al. 2015; Shen et al. 2025). Five trained observers surveyed each transect: two conducted direct visual searches and searched for signs within the 15 m strips on the left and right, respectively; one navigated along the centre line and measured perpendicular distances; one recorded GPS locations and terrain/habitat data and took photographs; and one followed at the rear to check for missed individuals or signs. The five observers remained within 10 m of one another and walked at a constant speed of 1-1.5 km/h. Observer roles were rotated amongst transects during different survey rounds to reduce observer bias (Buckland et al. 2001; Peres 1999). The fixed width of 30 m was selected because preliminary surveys indicated that direct-sighting and sign-detection rates for the target Macaca species were high and stable within 15 m on either side of the centre line. The narrow-strip method assumes that detection probability approaches one within the half-width and does not extrapolate density from distance; consequently, interpretation of the results is conservative (Karanth and Nichols 1998; Buckland et al. 2001). Sign data from the transects were used as evidence of distribution and activity, but were not included in estimates of troop size or number of individuals.

Signs were identified according to the morphological descriptions of Macaca species in A Guide to the Mammals of China (Smith and Xie 2008) and China’s Mammal Diversity and Geographic Distribution (Jiang et al. 2015). SXM and WCM exhibit identifiable differences in faecal diameter, length, shape and residual composition, as well as in the morphology and length-to-width ratios of the palm and toe pads in footprints. Two trained observers independently identified all signs. Signs that could not be reliably identified to species were recorded uniformly as “unidentified Macaca (Macaca sp.)” and excluded from species-specific analyses. All signs were photographed and measured and their GPS coordinates were recorded. Disagreements were reviewed by a third observer and signs that remained uncertain were excluded.

In this study, a “troop” was defined as a social unit composed of multiple individuals moving, foraging or resting together at close distances (≤ 50 m) within a given spatial and temporal range and exhibiting coordinated behavior (Peres 1999; Henderson 2002). During transect surveys, ≥ 2 individuals moving together or congregating within 50 m, with no observed troop fission, were counted as one troop. In camera-trap records, individuals appearing in the same frame or consecutively within 30 s, without clear spatial separation in the images, were counted as one troop. Records from the same camera station on the same day that were combined using the 30-min rule were treated as a single troop record. Simultaneous records from different camera stations or sites separated by ≥ 500 m were not combined automatically unless natural markings of individuals or evidence from the troop's age-sex composition indicated that they represented the same troop. Troop size was the maximum number of individuals that could be reliably identified in a single record and was not obtained by summing records.

Not all individuals were uniquely marked and neither spatial capture-recapture nor distance-sampling density estimation was used. Accordingly, the reported values represent the “minimum number of individuals observed” (MOI), rather than absolute population size (Peres 1999; Henderson 2002). MOI was calculated as follows: Independent individuals identified by natural markings in the combined camera-trap and transect datasets were deduplicated; repeat detections of the same troop were counted only once; troop size was taken as the maximum number of reliably identified individuals in a troop record; and individuals or troops of uncertain duplicate status were processed using the most conservative deduplication rule to avoid overestimation. Troop membership was determined from spatial synchrony (inter-individual distance ≤ 50 m), direction of movement and behavioral coordination. Troops recorded at different times or by different cameras or transects were considered the same troop only when naturally marked individuals matched or when troop size and age-sex composition were highly consistent and could be tracked spatially; otherwise, they were treated as independent troops. Camera traps and transect surveys were deployed concurrently from April to September 2025 to ensure temporal comparability and effective cross-validation of the two datasets.

## Results

### Evidence for Stable Distributions of the Two Macaque Species in Xizang 

The survey recorded 11 independent confirmed occurrence sites for SXM, all concentrated in the Lebu Valley area of Cona City, Shannan City, Xizang Autonomous Region (as shown by the diamond in Fig. 2). The area lies in a valley at 2,400-3,000 m on the southern slope of the Himalayas, has a subtropical humid montane monsoon climate and is covered by subtropical evergreen broad-leaved forest. Across the 11 occurrence sites, camera traps yielded seven independent SXM troop records, while four troops were directly observed during transect surveys (the infrared cameras and the SXM images captured by the transects in this survey are shown in Fig. 3. All sightings and camera-trap records included adults, subadults and infants, indicating a complete age structure in each troop. Transect surveys also recorded 13 signs of SXM, including faeces and footprints, at these sites. The signs were spatially continuous and occurred across multiple habitat types, including forest and shrubland.

According to the definition of “stable distribution” used in this study — a species has continuous or regular occurrence records within a specific area, together with populations capable of carrying out essential life activities, such as reproduction and foraging — the survey obtained the following concurrent evidence in the Lebu Valley area: (a) multiple spatially dispersed and independent occurrence sites (n = 11), indicating that the species was not merely an occasional visitor to the area; (b) multiple troop records that included infants, indicating normal reproductive activity by SXM in the area; and (c) spatially continuous sign records that corroborated the troops' activity ranges. Together, these results show that SXM has a continuous distribution in the Lebu Valley area and that populations capable of reproduction and other essential life activities are present.

The survey recorded 10 independent confirmed occurrence sites for WCM, mainly concentrated near Gangrigabu Mountain in Gedang Township and around the 80K locality in Damu Township, Medog County, Nyingchi City, Xizang Autonomous Region (as shown by the triangle in Fig. 2). This area lies east of the Yarlung Zangbo Grand Canyon at elevations of approximately 2,010-2,999 m. Vegetation is dominated by subtropical evergreen broad-leaved forest and mixed coniferous broad-leaved forest and the terrain is characterised by steep slopes and canyons (the infrared cameras and the WCM images captured by the transects in this survey are shown in Fig. 4).

Camera traps and transect surveys yielded 10 independent WCM troop records: six troops were photographed by camera traps and four were directly observed during transect surveys. Transect surveys also recorded nine WCM signs, including faeces and footprints. The sign locations were spatially clustered and matched the spatial pattern of the camera-trap records and direct sightings. According to the definition of “stable distribution” used in this study, the survey obtained the following evidence in Medog County: (a) 10 spatially independent occurrence sites covering two geographic sectors, Gedang Township and Damu Township; (b) infants in multiple troop records, indicating ongoing reproductive activity; and (c) continuous sign records around the occurrence sites, supporting regular activity by the species in these areas. These results indicate that WCM has a continuous distribution within Medog County and that populations capable of reproduction, foraging and other essential life activities are present.

### Troop Size and Social Structure of the Two Macaque Species

In this study, the sex of adult individuals was determined from two independent types of evidence: (1) direct visual identification by observers during field transect surveys, based on external genitalia, body size and facial sexual characteristics; and (2) camera-trap photographs or videos that clearly showed the genital region or typical maternal behavior, such as nursing or carrying an infant. Accordingly, all individuals observed carrying infants were classified as adult females. It should be emphasized that the number of infants provides a conservative lower bound for the number of adult females, rather than an exact estimate. This inference assumes that not every adult female was carrying an infant during the survey period, because some females were not in a reproductive period, had not conceived or had offspring that were already independent. The actual number of adult females may, therefore, exceed the number of infants recorded. In addition, sex determination of adults was constrained by image clarity, camera angle and individual posture and the sex of some individuals could not be determined reliably.

In the Lebu Valley area of Cona City, Shannan City, the survey recorded 11 independent SXM troops (Table 1). After the sizes of the troops—defined as the maximum number of individuals reliably identified in a single record—were aggregated, the SXM population in this area was estimated at 162-176 individuals, with a mean troop size of 14-16 individuals per troop. With respect to age structure, approximately 102-114 adults, 39-41 juveniles, and 21 infants were identified. Amongst adults whose sex could be determined, the adult male-to-female ratio was approximately 1:2. As this ratio was based on the subset of individuals whose sex could be determined and the number of infants provided only a lower bound for the number of adult females, the actual proportion of females in the population may be higher; uncertainty in the true sex ratio should, therefore, be acknowledged. The presence of infants indicates ongoing reproduction in each troop and supports the ability of the population to maintain recruitment within the survey area.

In the areas around Gangrigabu Mountain in Gedang Township and the 80K locality in Damu Township, Medog County, Nyingchi City, 10 independent WCM troops were recorded (Table 1). Population size was estimated at 154-171 individuals, with a mean troop size of 15-17 individuals per troop. The age structure comprised approximately 89-104 adults, 42-44 subadults and 23 infants. Amongst adults whose sex could be determined, the adult male-to-female ratio was approximately 1:2. This estimate was likewise limited by the success rate of sex determination and the infant count constituted only a lower bound for the number of adult females; uncertainty in the actual sex structure must, therefore, be noted. The widespread presence of infants also indicates that the WCM population has basic reproductive capacity.

## Conclusions

This systematic field survey obtained the first conclusive distribution records of SXM and WCM in the Xizang Autonomous Region and clarified their spatial distribution patterns and key habitat characteristics. SXM preferred montane Himalayan fir forests at elevations of 2,000-3,000 m, whereas WCM occurred widely in mid-elevation humid evergreen broad-leaved forests, deciduous broad-leaved forests and mixed coniferous broad-leaved forests. These results advance previous inferences, based on limited specimens or indirect information into a documented account of current distribution and provide a data foundation for subsequent population assessments and conservation planning.

The systematic implementation of this survey and the acquisition of precise distribution data for the two macaque species were made possible by recent improvements in field-monitoring conditions and advances in conservation work in Xizang. These distribution records reflect enhanced biodiversity conservation and monitoring capacity in the region and also suggest that, since the promulgation of the Wildlife Protection Law, populations previously overlooked because of limited survey coverage are being re-discovered and effectively protected. However, this study was based on a single year of surveys and, therefore, presents distribution ranges and relative abundance only as a current temporal snapshot; the data are insufficient to infer long-term temporal stability. Accordingly, the term “stable distribution” is strictly limited to the operational definition specified in the Methods: sustained records of demonstrable reproductive and foraging activity within the survey area, rather than a judgement about long-term population trends. The survey did not incorporate a time series or a comparative experimental design and any inference regarding population trends or attribution to external factors would exceed the available evidence.

Despite these limitations, the findings have clear conservation implications. Both macaque species are endangered, have relatively narrow distributions and face a real threat from habitat fragmentation. Their habitats also span the transition between subtropical and tropical climatic zones, making them ideal models for studying the evolution of high-elevation adaptation and socioecological strategies in primates. We therefore recommend prioritising the establishment of conservation areas or conservation sites within the core distribution areas so that an “umbrella effect” can benefit sympatric rare plants and animals and rapidly establish a long-term monitoring system that encompasses population dynamics, habitat quality and disturbance factors. These measures are both an urgent current conservation need and a regional reference for the conservation of montane primates worldwide; their effectiveness should be evaluated with subsequent monitoring data.

## Figures and Tables

**Figure 1.**
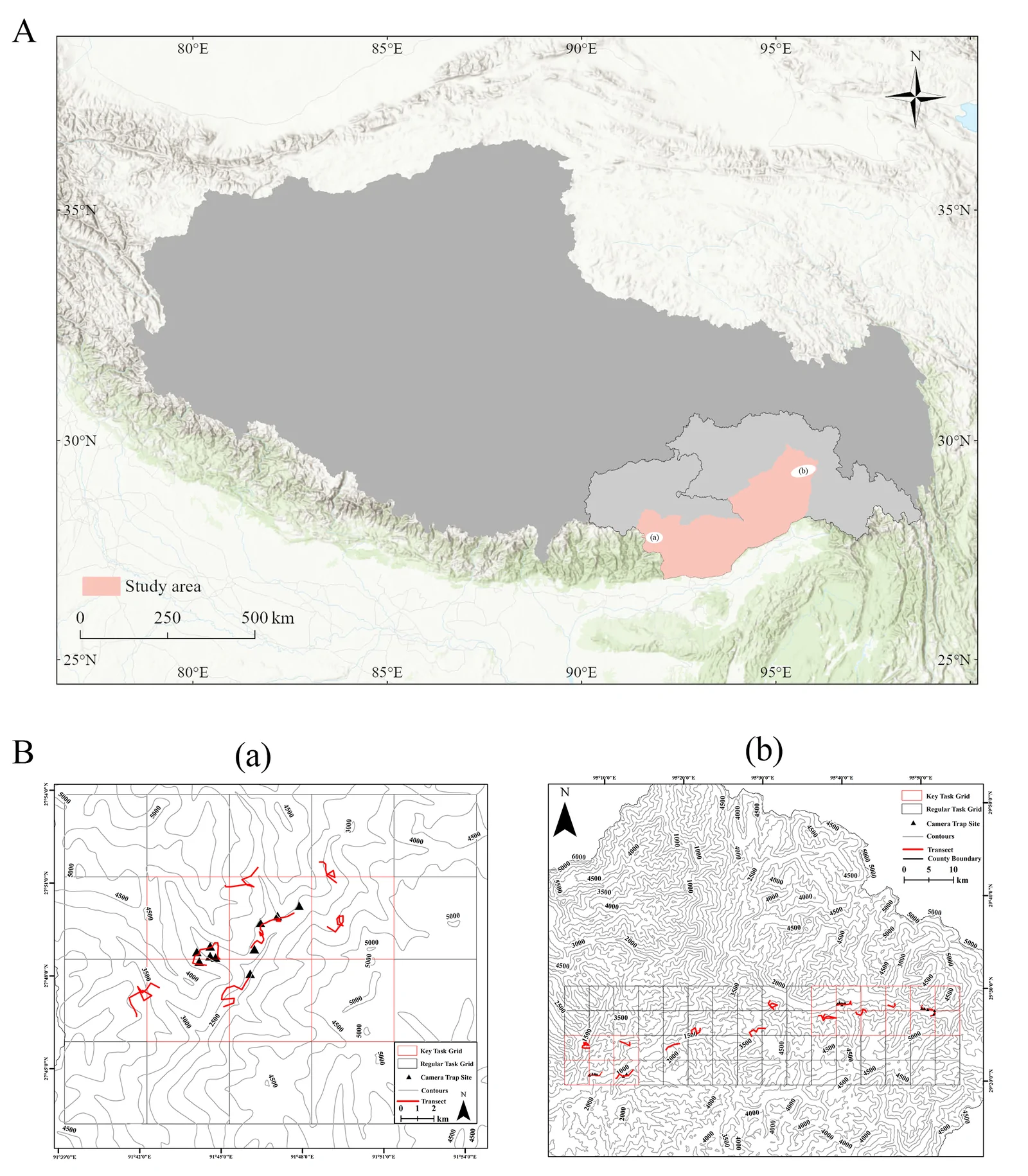
Study area map. **(A)** Geographic location of the study area; **(B)** Survey transects and camera trap locations. Red lines indicate survey transects and black triangles indicate camera trap deployment sites.

**Figure 2.**
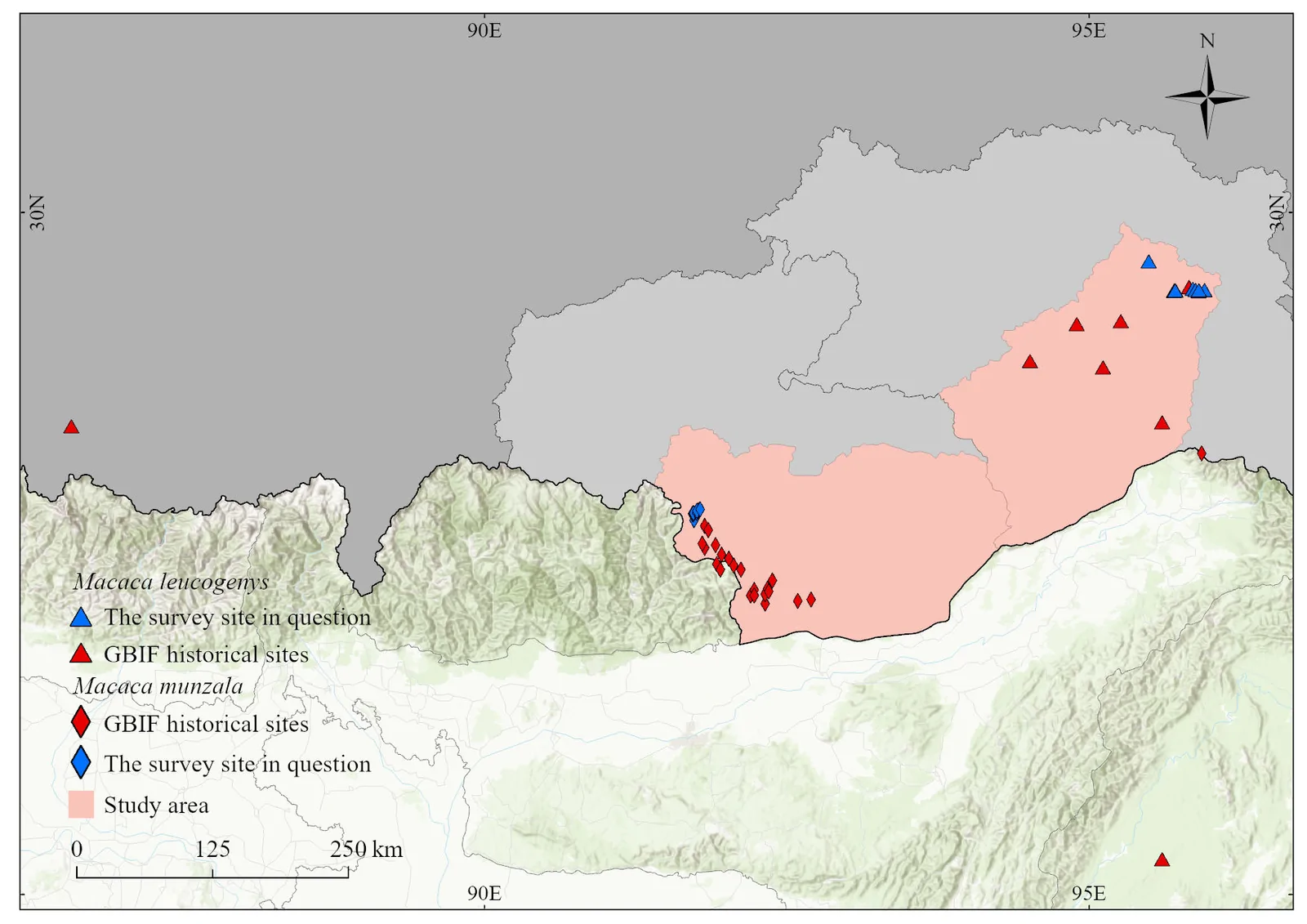
Schematic diagram of distribution sites of the two macaque species. Diamonds represent *Macaca munzala*, triangles represent *Macaca leucogenys*, red indicates historical GBIF occurrence records and blue indicates sites recorded in the present survey. Historical GBIF records are current as of April 2025.

**Figure 3.**
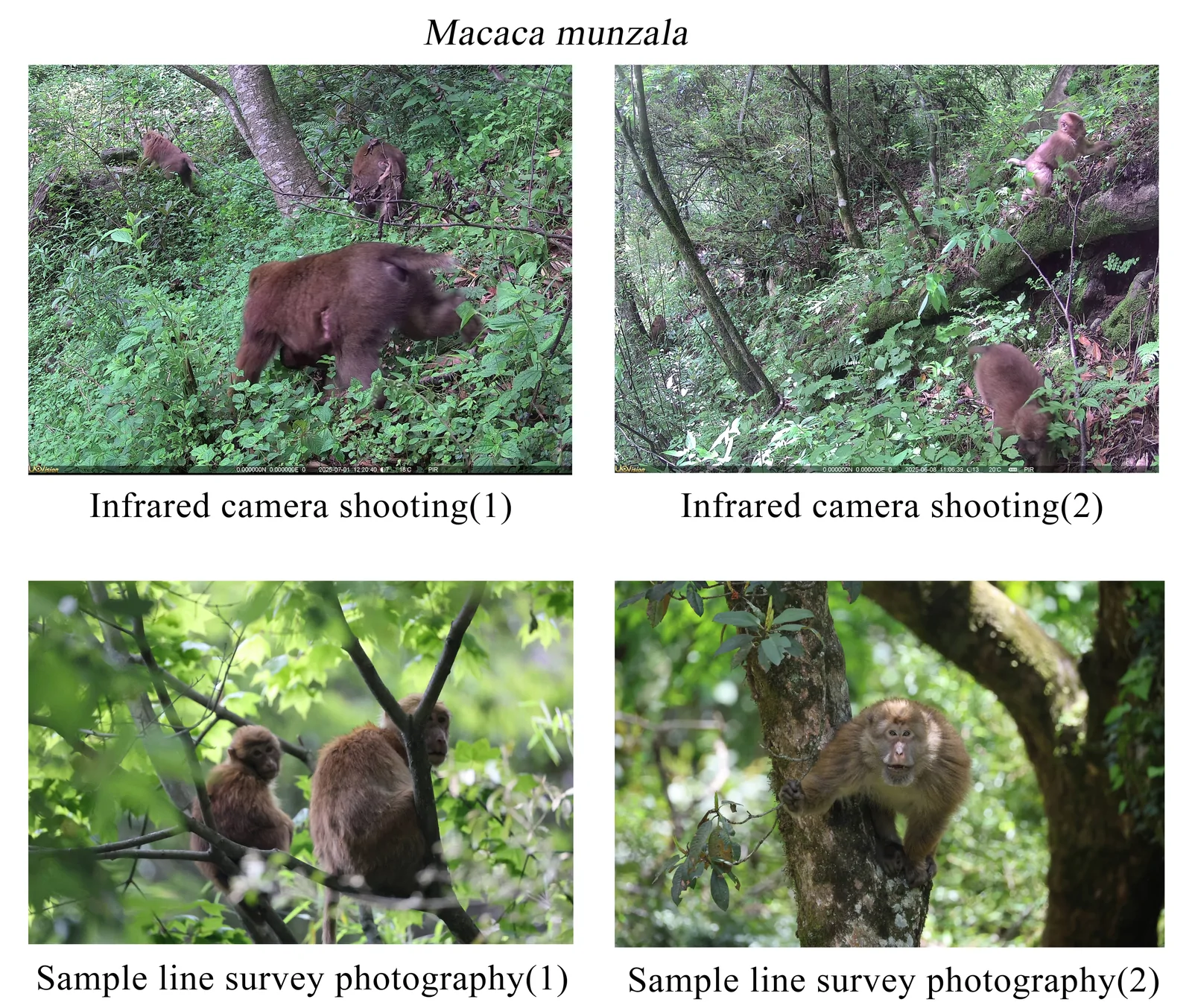
Photographs of *Macaca munzala* captured during the present survey.

**Figure 4.**
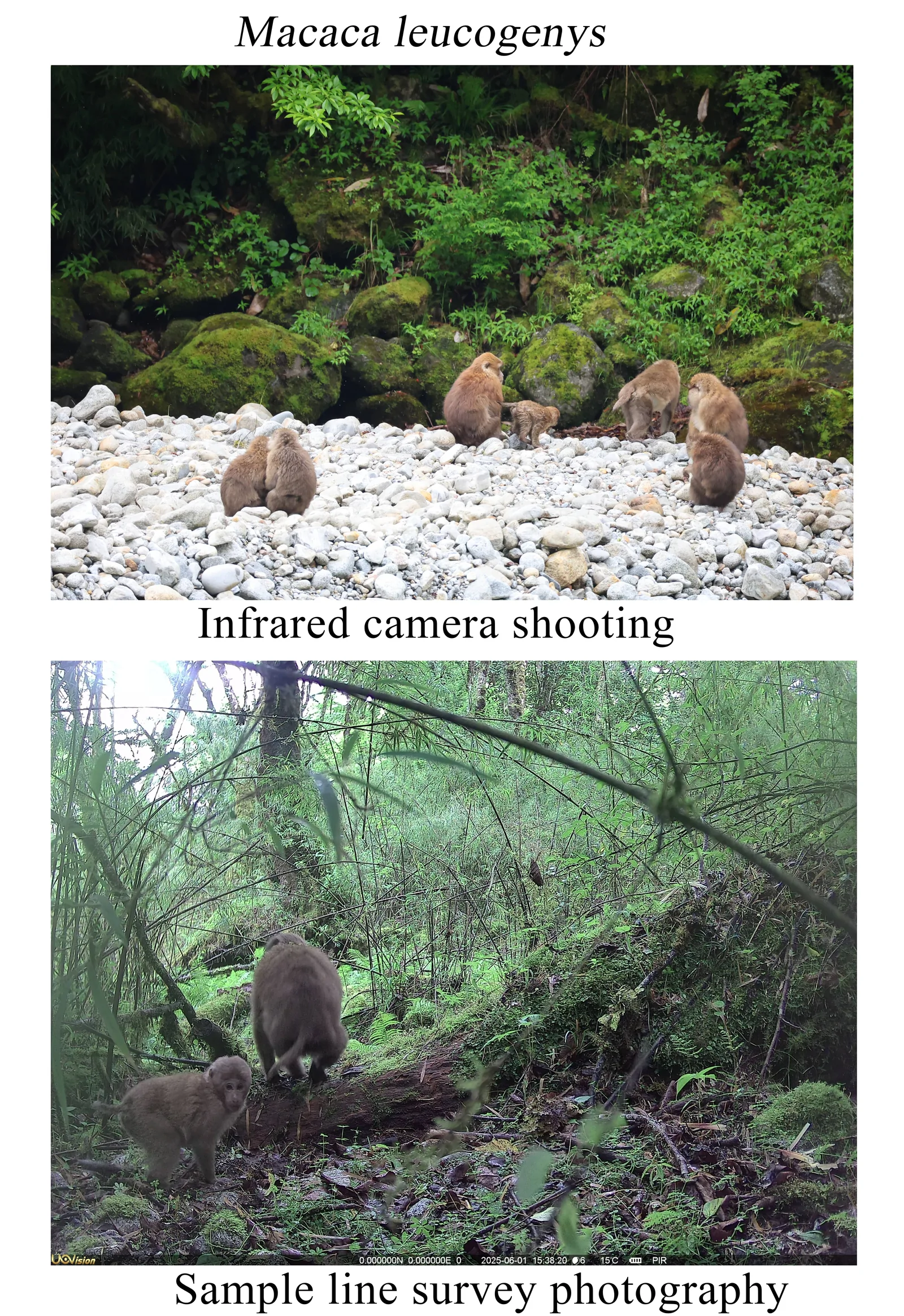
Photographs of *Macaca leucogenys* captured during the present survey.

**Table 1.** **Table 1. Population information for the Arunachal macaque and white-cheeked macaque in Xizang, China**.

Survey ID	Elevation	Location	Troop size				Total	Habitat type	Data source
			Adult males	Adult females	Juveniles	Infants			
Arunachal macaque									
1	2739 m	Cona City, Shannan City	2	6-8	3	2	13-15	Deciduous broad-leaved forest	Transect survey
2	2839 m		4	9-11	6	2	21-23	Deciduous broad-leaved forest	Transect survey
3	2467 m		3	12-14	5	1	21-23	Deciduous broad-leaved forest	Transect survey
4	2708 m		2-4	14	8	4	28-30	Deciduous broad-leaved forest	Transect survey
5	2411 m		2-4	12	6	4	24-26	Shrubland	Camera trap
6	3228 m		1	3	2	2	8	Deciduous broad-leaved forest	Camera trap
7	2953 m		3	6-8	4	3	16-18	Deciduous broad-leaved forest	Camera trap
8	2830 m		2	5	3	2	12	Shrubland	Camera trap
9	2868 m		2	10	2-4	1	15-17	Coniferous forest	Camera trap
10	2891 m			1				Coniferous forest	Camera trap
11	2934 m		3					Deciduous broad-leaved forest	Camera trap
White-cheeked macaque									
12	2565 m	Damu Township, Medog County	3	7	1	2	13	Montane evergreen broad-leaved forest	Transect survey
13	2010 m		2	4-6	4	3	13-15		Camera trap
14	2141 m	Gedang Township, Medog County	3	3-6	3	2	11-14		Transect survey
15	2746 m		1	1			2		Camera trap
16	2651 m		7	18	15-17	3	43-45		Camera trap
17	2133 m		3	6-7	2	3	14-15		Camera trap
18	2672 m		3	4-6	2	1	10-12		Camera trap
19	2999 m		2	4-7	2	2	10-13		Camera trap
20	2275 m		5	7-9	10	3	25-27		Camera trap
21	2265 m		2	4-6	3	4	13-15		Camera trap
